# Dysregulated post-translational modifications in granulosa cells drive ovarian dysfunction and potential infertility applications (Review)

**DOI:** 10.3892/ijmm.2026.5767

**Published:** 2026-02-16

**Authors:** Yufei Zhong, Yunfei Zou, Zhuoyuan Yang, Junjun Wang, Zezheng Pan, Jiugeng Feng

**Affiliations:** 1 Department of Neurosurgery, The First Affiliated Hospital, Jiangxi Medical College, Nanchang University, Nanchang, Jiangxi 330006, P.R. China; 2 College of HuanKui, Jiangxi Medical College, Nanchang University, Nanchang, Jiangxi 330006, P.R. China; 3 School of Queen Mary, Jiangxi Medical College, Nanchang University, Nanchang, Jiangxi 330006, P.R. China; 4 College of Second Clinical Medicine, Jiangxi Medical College, Nanchang University, Nanchang, Jiangxi 330006, P.R. China; 5 Jinggangshan Nashan Township Health Center, Jinggangshan, Jiangxi 343600, P.R. China; 6 Department of Basic Medical Sciences, Jiangxi Medical College, Nanchang University, Nanchang, Jiangxi 330006, P.R. China

**Keywords:** granulosa cells, post-translational modifications, follicular development, infertility, ovarian dysfunction

## Abstract

Ovarian granulosa cells (GCs), as key components of follicles, orchestrate follicular development and ovarian maturation through bidirectional communication with oocytes and through hormone synthesis. Their dysfunction substantially contributes to female infertility. Post-translational modifications (PTMs) carry out pivotal roles in the regulation of ovarian physiology and pathology by modulating GC proliferation, differentiation, apoptosis and steroid hormone secretion. The present review seeks to summarize the current advances in canonical PTMs such as phosphorylation, methylation, acetylation and ubiquitination, as well as novel protein modifications such as SUMOylation and lactylation, particularly focusing on their roles in the proliferation, differentiation and apoptosis of GCs at the molecular level. Moreover, the present review explores how aberrant PTMs impair GC function, leading to follicular developmental disorders, and proposes that targeting PTM-regulated signaling in GCs may provide novel therapeutic strategies for ovarian dysfunction. Collectively, the present review aims to provide insights into elucidating the etiology of infertility, and establishing a theoretical foundation for the development of PTM-targeted reproductive interventions.

## 1. Introduction

### Granulosa cells (GCs), dysfunction and infertility

GCs are the specialized somatic cells of the sex cord-stromal lineage found within the mammalian ovarian follicle, where they typically form either a monolayer or a multilayer around the oocyte to facilitate follicular development and steroid hormone synthesis (1). During follicular development, GCs differentiate into two functionally distinct subpopulations: Mural GCs, which align with the basal lamina to constitute the follicular wall, and cumulus cells, which maintain communication with the central oocyte (2). These cells also establish gap junction-mediated communication with the oocyte, providing essential nutrient support and regulating the microenvironment necessary for oocyte maturation (3,4). The proper proliferation and differentiation of GCs are imperative for the correct formation of follicles and the subsequent quality of the embryo, both of which are key determinants of female fertility (5). Infertility has increasingly been acknowledged as a considerable global public health issue. Female factors contribute to >50% of infertility cases, predominantly driven by environmental degradation and adverse lifestyle conditions (6). Dysfunctions in GCs include a range of pathophysiological processes, such as imbalances in proliferation and differentiation, abnormalities in cellular senescence and apoptosis, and disturbances in hormone synthesis (7,8). The mechanisms contributing to these dysfunctions are complexly associated with excessive oxidative stress, mitochondrial dysfunction, abnormal inflammatory responses and endoplasmic reticulum stress (ERS) (8-11). Such dysfunctions can markedly compromise female fertility through various molecular pathways, including the premature depletion of the primordial follicle pool (12), disrupted folliculogenesis (13), ovulation disorders (14) and embryo implantation failures (15). Given the pivotal role of GCs in folliculogenesis, a comprehensive understanding of their regulatory mechanisms is indispensable. Acquiring this knowledge is key for identifying potential biomarkers for the early diagnosis of infertility and could lay the groundwork for the development of GC-targeted therapeutic strategies, thereby advancing precision medicine in the management of clinical infertility.

### Protein post-translational modifications (PTMs) regulate GC characterization

PTMs of proteins refer to the process of adding or removing chemical groups from amino acid residues in the polypeptide chains of proteins. These modifications, defined as the side chain modification of amino acids that occur after protein synthesis (16,17), provide a powerful means to augment and regulate protein function. PTMs are essential cellular mechanisms that regulate protein activity, stability and subcellular localization through reversible covalent modifications. As fundamental regulatory mechanisms, PTMs carry out a pivotal role in orchestrating diverse biological processes (18). Within GCs, PTMs meticulously regulate cellular proliferation, differentiation, apoptosis and hormone secretion by modulating signaling pathways, epigenetic landscapes, proteostasis and metabolic modifications (7,19,20). Canonical PTMs, including phosphorylation, methylation, ubiquitination and acetylation, are important in regulating the functions of GCs. For example, PTM-mediated activation of the PI3K/AKT/FOXO3 signaling pathway is essential for primordial follicle activation, underscoring the fundamental role of PTMs in folliculogenesis (21). Additionally, dynamic histone modifications, particularly H3K4me3, H3K9me and H3K27me3, act as epigenetic switches that precisely regulate progesterone production during luteinization (20). Advancements in high-resolution mass spectrometry have revealed several novel PTMs including lactylation (22), crotonylation (23), neddylation (24), lysine succinylation (25) and lysine β-hydroxybutyrylation (26), which notably influence GC. Accumulating evidence suggests that aberrant PTM patterns in GCs are a principal factor contributing to infertility. Thus, elucidating the PTM profiles in GCs not only enhances the understanding of the pathogenesis of infertility but also aids in the development of therapeutic strategies targeting specific PTM enzymes.

### Clinical perspectives on PTM-targeted interventions in GCs

Assisted reproductive technology (ART) serves as an important intervention for fertility preservation. Dysfunctional GCs impair oocyte quality, maturation and embryo viability, thereby reducing the success rates of ART (5,27,28). Almeida *et al* (5) suggested that a pre-ART evaluation of GC quality could enhance the selection of oocytes and embryos. Notably, ovarian hyperstimulation syndrome (OHSS), a severe complication of ovarian stimulation during ART, may benefit from therapeutic approaches involving PTMs in the future. Zheng *et al* (29) demonstrated that melatonin mitigates reactive oxygen species (ROS)-induced apoptosis in GCs via the phosphorylation regulation of the SESN2-AMPK-mTOR axis, suggesting considerable clinical potential for OHSS therapy. Furthermore, a clinical study on polycystic ovary syndrome (PCOS) indicated that Myo-Inositol supplementation improves steroidogenesis, oocyte maturation, fertilization rates and embryo quality through phosphorylation-dependent modulation of the ERK1/2 and AKT pathway in cumulus cells (30). Concurrently, Zhang *et al* (31) confirmed that electro-acupuncture improves ovarian function in a premature ovarian failure (POF) mouse model through phosphorylation-mediated regulation of the PI3K/AKT/mTOR signaling cascade. Collectively, these studies underscore that PTM-targeted interventions in GCs represent a promising therapeutic approach for treating female infertility.

However, current reviews on PTMs in female infertility focus on physiological processes such as folliculogenesis (32), oocyte meiotic maturation and embryonic development (33,34) or are restricted to one specific pathological context, such as POF (35), unexplained recurrent pregnancy loss (36), recurrent spontaneous abortion (37), PCOS (38,39) and endometriosis (40). Although PTMs have been confirmed to be involved in GCs, the majority of studies are fragmented, focusing largely on a single class of modifications, without integrating how different PTM pathways interact (41-43). Comprehensive elucidation of the dynamic interplay between the full spectrum of PTMs and GC physiology remains limited. From the perspective of GCs, the present review integrates studies on how various PTMs regulate diverse vital activities of GCs, including proliferation, differentiation, apoptosis and steroid hormone secretion, evaluates their potential for therapeutic targeting and ultimately aims to establish a novel framework for understanding the regulatory networks of GCs, thereby facilitating PTM-based clinical interventions for infertility.

## 2. Core regulatory networks of PTMs on the fate of GCs

### Phosphorylation (Table I) (41,43-74)

Phosphorylation is one of the most common PTMs of proteins. Protein phosphorylation refers to the addition of a phosphate group to proteins, mainly serine, threonine and tyrosine, and activates/inactivates numerous enzymes and receptors through phosphorylation and dephosphorylation to regulate the function and localization of proteins, which is an important cellular regulatory mechanism (75). Protein phosphorylation serves as a fundamental regulatory mechanism in folliculogenesis. This reversible modification dynamically controls the fate of GCs during follicular development through precisely regulated signal transduction cascades. Dysregulated phosphorylation disrupts key physiological processes in GCs, including proliferation, autophagy, differentiation, apoptosis and hormone secretion. It also impairs the bidirectional metabolic exchange between GCs and oocytes, ultimately leading to aberrant follicular atresia and diminished female fertility (21,76).

The regulation of GC proliferation is meticulously governed by phosphorylation-dependent signaling pathways, notably the ERK, Hippo/YAP1 and EGFR/PI3K/AKT/mTOR pathways (77). For example, FGF12 has been shown to promote GC proliferation by enhancing the phosphorylation of ERK1/2 (44). Additionally, the mitochondrial proteins MIGA1/2 support the proliferation of GCs through dual mechanisms that involve the phosphorylation of both AKT and Hippo/YAP1 signaling, specifically through the phosphorylation of YAP1 at Ser127 (49). Elevated levels of ROS induce pathological oxidative stress, leading to disruptions in mitochondrial homeostasis and ERS, which are pivotal in driving apoptosis in GCs. It was demonstrated that exposure to ROS in mouse GCs decreases phosphorylation at Ser637 while increasing phosphorylation at Ser616 on the mitochondrial fission protein Drp 1, a dysregulated phosphorylation pattern that exacerbates mitochondrial dysfunction and ultimately triggers oxeiptosis in GCs (73). Xue *et al* (43) found that an imbalance in ROS suppresses AKT phosphorylation, deactivating the PI3K/AKT pathway and initiating autophagy via reduced mTOR inhibition. Additionally, GLP-1/GLP-1R signaling influences GC proliferation and apoptosis through the phosphorylation of FOXO1 at both Ser256 and Ser319 (66). Concurrently, excessive ERS triggers reticulophagy as a compensatory response, mitigating GC apoptosis-mediated follicular atresia in GCs (78).

Progesterone (P4) carries out a key role in embryonic development and uterine implantation. Large luteal cells, which differentiate from GCs, synthesize P4 using cholesterol as the substrate (71,79). Luteinizing hormone (LH) enhances the efficiency of cholesterol mobilization through a dual regulatory mechanism mediated by the protein kinase A signaling pathway, which phosphorylates both AMP-activated protein kinase and hormone-sensitive lipase to optimize P4 biosynthesis (71,72,80). The production of P4 induced by LH remains unaffected by the mTOR inhibitor rapamycin, suggesting that LH may regulate P4 synthesis via non-canonical molecular pathways; thus, the precise mechanisms remain to be elucidated (72).

In summary, aberrant phosphorylation modifications within key signaling pathways contribute to the dysfunction of GCs. Some targeted inhibitors, such as the mTOR inhibitor rapamycin (77) and the ERK pathway antagonist ISRIB (44), have proven effective in restoring GC function. Consequently, the development of precision therapeutics that modulate GC phosphorylation networks offers a promising novel strategy. Future research should focus on the rational design of drugs that utilize established phosphorylation regulatory networks, coupled with a comprehensive mapping of phosphorylation dynamics across various stages of follicular development (43).

### Methylation (Table II) (15,81-98)

Protein methylation is an important PTM that occurs primarily on lysine and arginine residues and modulates histone and non-histone functions (99,100). Methylation represents a key determinant of the fate of GCs. During luteinization, the LH surge induces chromatin remodeling through histone methylation, thus orchestrating GC luteinization and hormone synthesis, including P4 and estrogen. Histone modifications such as trimethylation of lysine 4 on histone H3 (H3K4me3) and lysine 36 on histone H3 generally facilitate transcriptional activation, while trimethylation of lysine 9 on histone H3 (H3K9me3) and lysine 27 on histone H3 (H3K27me3) are associated with transcriptional repression (91,101). The enzymes StAR and CYP11A1 are key in P4 synthesis. Following the LH surge, the ERK1/2 signaling pathway is activated, increasing methylation at the H3K4me3 site within the promoter regions of these genes, while concurrently reducing methylation at the H3K9me3 and H3K27me3 sites (20,82,86). This coordinated alteration enhances transcriptional activation and supports the expression of steroidogenic genes. Concurrently, the sustained expression of lysine-specific demethylase 1A (LSD1), an H3K4 demethylase, ensures precise epigenetic regulation over the P4 biosynthesis pathway (87). Moreover, modifications of H3K9me3 and H3K27me3 carry out roles in regulating reproductive physiology by maintaining normal estrous cycles and follicular recruitment through the regulation of the inhibin α promoter (15,88), and by promoting luteal angiogenesis via vascular endothelial growth factor transcriptional regulation (89). Beyond P4 regulation, histone methylation also controls estrogen secretion by modulating the expression of CYP19A1, the rate-limiting enzyme in estrogen synthesis, through modifications such as H3K4me3 and H3K9me3 (83-85). Specifically, dysregulation of H3K9me3 at the *Cyp19a1* gene locus may disrupt endocrine function and contribute to the pathogenesis of endometriosis (84). Collectively, these findings highlight that histone methylation acts as a bidirectional epigenetic switch, dynamically balancing transcriptional activation and repression to precisely regulate luteal function and reproductive homeostasis.

Aberrant elevations of H3K36me1/2/3 and H3K27me3 have been demonstrated to markedly impair the proliferative capacity of GCs and promote their apoptosis (90,91). Conversely, reduced levels of H3K4me2/3 induce G2/M phase cell cycle arrest (94). Moreover, deficiencies in KDM4B/5B/5C demethylases lead to the accumulation of DNA double-strand breaks, resulting in S phase arrest and promoting apoptosis in GCs (95-97). During ovarian aging, decreased activity of methyltransferase is associated with the dysregulated redistribution of H3K9me2/3 and H3K4me2/3, suggesting that aberrant PTMs serve as one of the fundamental drivers of reproductive senescence (92,93,102).

In addition to modifications involving lysine methylation, arginine methylation also carries out a key role in the developmental regulation of GCs. Protein arginine methyltransferase 5 (PRMT5), the major type II enzyme, is responsible for the symmetric dimethylation of arginine, which facilitates internal ribosome entry site (IRES)-dependent translation of WT1 mRNA by methylating HnRNPA1 (98,103). In PRMT5-deficient GCs, the expression of steroidogenic genes was overactivated by WT1 downregulation. This dysregulation drives the premature differentiation of GCs into a luteinized-like state. Such precocious differentiation disrupts the essential physical and nutritional support between GCs and the oocyte, ultimately leading to follicular developmental arrest, structural disorganization, atresia and female infertility (98,104). Clinically, the repressive chromatin in mural GCs exhibits considerable enrichment of H3K27me3 methylation during folliculogenesis in patients with diminished ovarian reserve, validating the pivotal role of histone methylation in determining the fate and function of GCs (105).

### Acetylation (Table III) (15,20,85,106-117)

Protein acetylation can be broadly categorized into two types: Histone acetylation and non-histone acetylation. Both types involve the transfer of acetyl groups to lysine residues via an enzyme-catalyzed reaction (118). This reversible modification is tightly regulated by lysine acetyltransferases, histone deacetylases (HDACs) and the sirtuin family of deacetylases (119).

**Figure 1:**
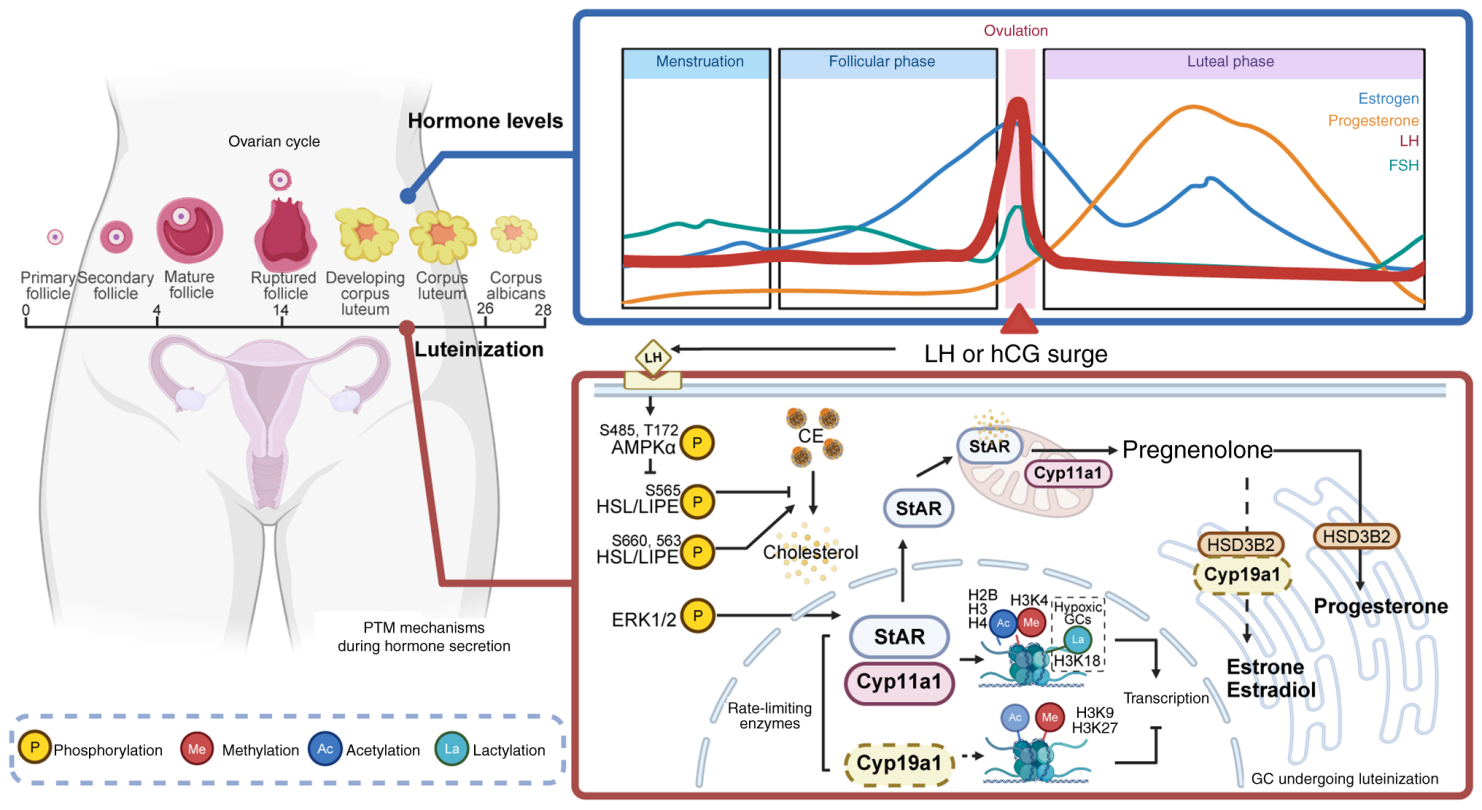
PTMs regulate steroid hormone synthesis during luteinization of GCs following the LH/hCG surge. The LH/hCG surge triggers ovulation and luteinization, processes critically dependent on PTMs to regulate steroid hormone secretion. HSL/LIPE releases cholesterol from CE, a step activated by LH signaling via AMPKα to alleviate inhibitory phosphorylation. Progesterone synthesis is promoted by ERK1/2-driven histone PTMs (acetylation, methylation and lactylation), which activate transcription of StAR and Cyp11a1. Conversely, estrogen synthesis is suppressed by inhibiting Cyp19a1 expression via repressive histone methylation and diminished acetylation. (Created in BioRender.com). PTM, post-translational modification; GCs, granulosa cells; hCG, human chorionic gonadotropin; LH, luteinizing hormone; CE, cholesterol ester; StAR, steroidogenic acute regulatory protein; HSL, hormone-sensitive lipase; LIPE, lipase E, gene encoding hormone-sensitive lipase; FSH, follicle-stimulating hormone; AMPK, AMP-activated protein kinase.

During human Chorionic Gonadotropin (hCG)-induced ovulation, H3K27ac rapidly undergoes deacetylation within 1 h, followed by a re-establishment of acetylation at elevated levels across specific genomic regions. This 'erase-and-rewrite' kinetic pattern is essential for the activation of transcription in genes associated with ovulation (106). Consequently, targeting this dynamic process of erasure and re-establishment offers notable therapeutic potential for the treatment of ovulatory disorders and merits further exploration. In the context of steroidogenesis, butyric acid promotes the acetylation of H3K9 by inhibiting HDACs, while the activity of HDACs themselves synergistically augments hormone production through dual stimulation of the PPARγ/PGC1α pathway (107). Notably, exposure to nicotine leads to the enrichment of HDAC3 at the cyclooxygenase 1 (COX1) promoter region in GCs, resulting in histone hypoacetylation that suppresses COX1 transcription and consequently inhibits PGE2 biosynthesis. These nicotine-induced epigenetic modifications trigger apoptosis and autophagy in GCs, ultimately impairing follicular maturation (108). As depicted in Fig. 1, beyond methylation, the dynamic alterations in histone acetylation are important in pre-ovulatory GCs. Upon LH induction, the dynamic acetylation of H2BK5, H3K9 and H4 facilitates chromatin remodeling, thereby regulating the expression of CYP19A1 and inhibin α and coordinating key processes such as estrogen synthesis, follicular development and ovulation (15,20,85,109). Moreover, the transcription of StAR, a rate-limiting enzyme in P4 synthesis, is dynamically regulated through acetylation of H3 and H4 at its promoter during this period (20). Thus, histone acetylation carries out a pivotal role in gene expression by mediating dynamic changes in chromatin structure, serving as a fundamental regulator in physiological processes including steroidogenesis, folliculogenesis and ovulation.

In addition to modifications in histone acetylation, previous studies have unveiled a key role for non-histone protein acetylation in various key cellular processes in GCs, such as proliferation, apoptosis, hormonal signaling, DNA methylation, cell cycle progression, autophagy and metabolism (110-112,116,120,121). Silencing information regulator 2 related enzyme 1 (SIRT1), an NAD^+^-dependent deacetylase, deacetylates FOXO1 and regulates its activity, especially under conditions of stress (112,122). This dysregulation facilitates the nuclear translocation of FOXO1, leading to the activation of pro-apoptotic genes, such as *Puma*, *Bim*, *Trail* and *Fas ligand*, and thus inducing apoptosis in GCs (112,113,123). Conversely, the activation of SIRT1-mediated deacetylation has been shown to rescue GCs from oxidative stress through the JNK/FOXO1 signaling pathway (113,124). Another study has shown that acetylation of FOXO1 in GCs is associated with ovarian competence. A decrease in FOXO1 acetylation levels enhances apoptosis in GCs, thereby increasing meiotic defects and aneuploidy in oocytes (114). These findings collectively highlight the dual role of FOXO1 acetylation in stress adaptation, although further research is needed to elucidate the mechanisms underlying this dual functionality.

In addition to FOXO1, the tumor suppressor protein p53 also carries out a pivotal role in promoting GC apoptosis, particularly under conditions of oxidative stress. SIRT1 inhibits p53 activity through deacetylation at the K382 site, and its downregulation leads to the accumulation of acetylated p53, thereby exacerbating H_2_O_2_ or TNF-α induced apoptosis in GCs (115,116). Furthermore, the inhibition of T-LAK cell-originated protein kinase amplifies this pathway by diminishing SIRT1 expression, resulting in sustained activation of p53 acetylation and subsequent caspase-dependent apoptotic cascades (117). Although strategies targeting SIRT1 activation (for example, NAD^+^ boosters) or modulation of p53 acetylation (for example, HDAC inhibitors) have demonstrated potential in protecting ovarian function, a comprehensive evaluation of their specificity and potential off-target effects remains essential (117).

In summary, acetylation modifications targeting both histone and non-histone proteins play integral roles in regulating GC proliferation, hormonal synthesis and follicular development. Dysregulation of acetylation is associated with infertility, presenting promising therapeutic targets for related reproductive disorders.

### Ubiquitination (Table IV) (125-134)

Ubiquitination is a broad PTM that falls into two main types, called monoubiquitin and polyubiquitin, whose states are regulated by ubiquitination and deubiquitination systems, usually triggering degradation through proteasome and autophagy pathways. The coordinated action of two predominant protein degradation pathways is key for GC differentiation, steroidogenesis and follicular development (135,136).

A study demonstrated that the E3 ligase SYVN1 suppresses mitochondrial fission and apoptosis, and delays follicular atresia by targeting Drp 1 for degradation (125). Ma *et al* (126) discovered that *Cry1* deficiency impairs NCOA4 degradation due to the downregulation of the E3 enzyme HERC2, which triggers iron overload and senescence via the ferritin-lysosome pathway. Additionally, USP14, the deubiquitinating enzyme (DUB) impairs DNA repair mechanisms through its deubiquitination activity, contributing to the pathogenesis of POF (127). By contrast, another DUB, UCHL1 promotes follicular development by stabilizing voltage-dependent anion channel 2, which enhances cholesterol transport and estradiol synthesis (128).

Moreover, ubiquitination is also involved in the regulation of metabolic and stress response pathways. Liu *et al* (129) found that SKP2-mediated ubiquitination of the glycolytic enzyme phosphoglycerate kinase 1 (PGK1) stabilizes the androgen receptor, thereby associating aberrant glucose metabolism with ovulation disorders in PCOS. Similarly, neuronal precursor cells expressed developmentally down-regulated 4-like (NEDD4L) directly promotes glutathione peroxidase 4 ubiquitination, inducing ferroptosis in GCs, a process considerably exacerbated in PCOS and ultimately leads to follicular dysfunction (130). Regarding autophagy regulation, GCs resist apoptosis through two distinct mechanisms: Deubiquitination-dependent stabilization of TGFβR2/SMAD4 signaling or melatonin-induced proteasomal degradation of BimEl (133,134).

Previously, therapeutic strategies targeting ubiquitinating enzymes have shown promising potential for improving ovarian function. Zhang *et al* (132) revealed that primordial follicular activation peptide 1 (PFAP1) stabilizes minichromosome maintenance complex component 5 to promote primordial follicle activation. Furthermore, lentiviral overexpression of the E3 ligase Peli1 in regulatory T cells enhances GC survival and promotes recovery of ovarian function, offering a novel approach for the treatment of POF (137).

To summarize, E3 ligases and DUBs act as molecular switches that finely regulate GC responses to hormonal and environmental signals. The aforementioned studies demonstrate that ubiquitination modification serves as a fundamental regulatory mechanism essential for maintaining proteome stability and signal transduction within GCs, offering novel strategies and therapeutic avenues for ovarian diseases.

### Novel PTMs (Table V) (19,22,23,25,138)

Beyond the four canonical PTMs previously discussed, recent research has identified several novel PTMs that carry out key roles as regulators in steroidogenesis, cell proliferation and apoptosis (22,25,139,140). The dysregulation of these PTMs has been associated with ovarian pathologies.

Lactylation, a protein modification induced by lactate accumulation under conditions of hypoxic or metabolic stress, carries out a pivotal role in the regulation of GCs (141). Wu *et al* (22) demonstrated that hypoxia accelerated hCG-induced GC luteinization, which could be inhibited by blocking lactate production or lactylation. Mechanistically, hCG selectively increases H3K18la, thereby augmenting the transcription of CYP11A1 and STAR, and consequently stimulating P4 production during GC luteinization. Additionally, the non-histone protein CREB at K136 has been identified as a potential lactylation site that mediates hCG-induced luteinization, which may activate proliferative signaling pathways and contribute to GC function and survival (22,55).

Crotonylation, first identified in 2011 (142), shares certain enzymatic systems and targets with acetylation, employing crotonyl-CoA as a substrate to transfer crotonyl groups onto lysine residues. This modification is involved in key cellular processes including metabolism, cell cycle regulation and cellular organization (143,144). Zhou *et al* (23) revealed that ANXA2cr enhanced its interaction with the EGFR, promoting EGFR endocytosis and subsequent phosphorylation. This modification regulates the proliferation and apoptosis of cumulus cells, thereby affecting the meiotic resumption and maturation of oocytes.

Neddylation is a PTM in which the ubiquitin-like protein neural precursor cell expressed developmentally downregulated protein 8 is covalently conjugated to target proteins via a dedicated enzymatic cascade. This process regulates fundamental cellular functions, most notably by activating Cullin-RING Ligases E3 (CRL) and thereby controlling protein stability and signaling (145). Chen *et al* (19) found that MLN4924-mediated inhibition of Cullin protein neddylation disrupts the activity of the CRL complex and downregulates the expression of PPARα/γ. This results in the suppression of anti-apoptotic genes while paradoxically activating proliferative pathways in GCs. Notably, MLN4924 also inhibits the neddylation of enzymes involved in lipid synthesis, leading to disrupted energy metabolism. These dual effects suggest its potential utility as a therapeutic target for ovarian protection.

Research has also clarified that O-GlcNAcylation and lysine succinylation (Ksuc) are not merely apoptotic regulators but pivotal modulators of GC physiology. O-GlcNAcylation occurs through the attachment of an O-GlcNAc group to serine or threonine residues on protein substrates, dynamically controlled by O-linked β-N-acetylglucosamine transferase and O-GlcNAcase (146). Disruption in O-G lcNAc modification homeostasis impairs energy metabolism in GCs, affecting glycolysis, mitochondrial function and the tricarboxylic acid cycle, ultimately leading to cellular dysfunction and apoptosis (138). Concurrently, succinylation occurs by the transfer of succinyl groups from succinyl-CoA to amino acid residues of the protein to be modified by succinyltransferases, with lysine being the most easily-modified amino acid. Le *et al* (25) identified Ksuc as a potential driver of ovarian aging, revealing that aberrant Ksuc accumulation compromises ovarian reserve markers, (such as anti-Mullerian hormone (AMH) and estrogen (E2), and promotes apoptosis through the upregulation of the aging marker P21. Together, these emerging PTMs represent promising biomarkers for assessing ovarian quality and offer novel therapeutic targets for fertility preservation.

## 3. Crosstalk of PTMs

The regulation of GCs involves not only individual PTMs but also crosstalk and synergistic interactions among various modifications, which together form a dynamic regulatory network within GCs.

### Phosphorylation + acetylation crosstalk

Phosphorylation and acetylation demonstrate synergistic effects in the modulation of AMH expression. The histone acetyltransferase p300 is dually activated through PI3K/AKT-mediated phosphorylation and direct interaction with SMAD2/3. These cooperative interactions considerably enhance H3K27ac, promoting AMH expression, which is important for restricting the recruitment of primordial follicles and maintaining the ovarian reserve. However, this activation can be counteracted by the recruitment of HDAC2 induced by follicle-stimulating hormone, creating a dynamic regulatory balance in GCs, thereby strictly regulating the timing of follicle selection and preventing premature ovarian exhaustion (147).

### Phosphorylation + demethylation crosstalk

Fibroblast growth factor 9 (FGF9) functions as a key regulator of follicular kinetics and GC proliferation (148). The efficacy of FGF9 signaling is contingent upon the levels of histone H3K4me2 in GCs. An enrichment of H3K4me2 enhances FGF9-mediated proliferation and steroidogenesis in GCs, whereas its depletion results in the downregulation of FGF9, which consequently triggers GC differentiation and follicular selection. This epigenetic regulation is mediated by LSD1, which undergoes considerable enhancement following phosphorylation (149).

### Crotonylation + phosphorylation crosstalk

Cumulus cells, specialized GCs connected with oocytes by transzonal projections, form a structure known as the cumulus-oocyte complex (150). A study showed that increased lysine crotonylation of ANXA2 enhances its binding to EGFR, thereby activating the EGFR pathway. This activation triggers a subsequent phosphorylation cascade, modulating the phosphorylation of AKT and ERK, which in turn promotes cumulus cell proliferation and suppresses apoptosis, ultimately supporting cumulus cell-dependent maturation and influencing oocyte maturation (23).

### Acetylation + methylation + phosphorylation crosstalk

Several studies have shown that palmitic acid (PA) induces ERS and even causes apoptosis in GCs (151,152). Shibahara *et al* (153) discovered that PA induces apoptosis in GCs through a mechanism involving triple PTMs. Specifically, the inhibition of AKT Ser473 phosphorylation by PA leads to the suppression of the PI3K/AKT pathway, which in turn activates apoptotic effectors such as caspase-3. Additionally, PA treatment results in the accumulation of ceramide in GCs, which directly inhibits cell proliferation. In oocytes, PA suppresses the AKT signaling pathway while concurrently upregulating the activities of histone deacetylase and methyltransferase. These alterations lead to hypoacetylation at H4K12 and hyperdimethylation at H3K9, culminating in epigenetic dysregulation that compromises oocyte quality.

## 4. Potential clinical applications of GC-associated PTMs

**Figure 2:**
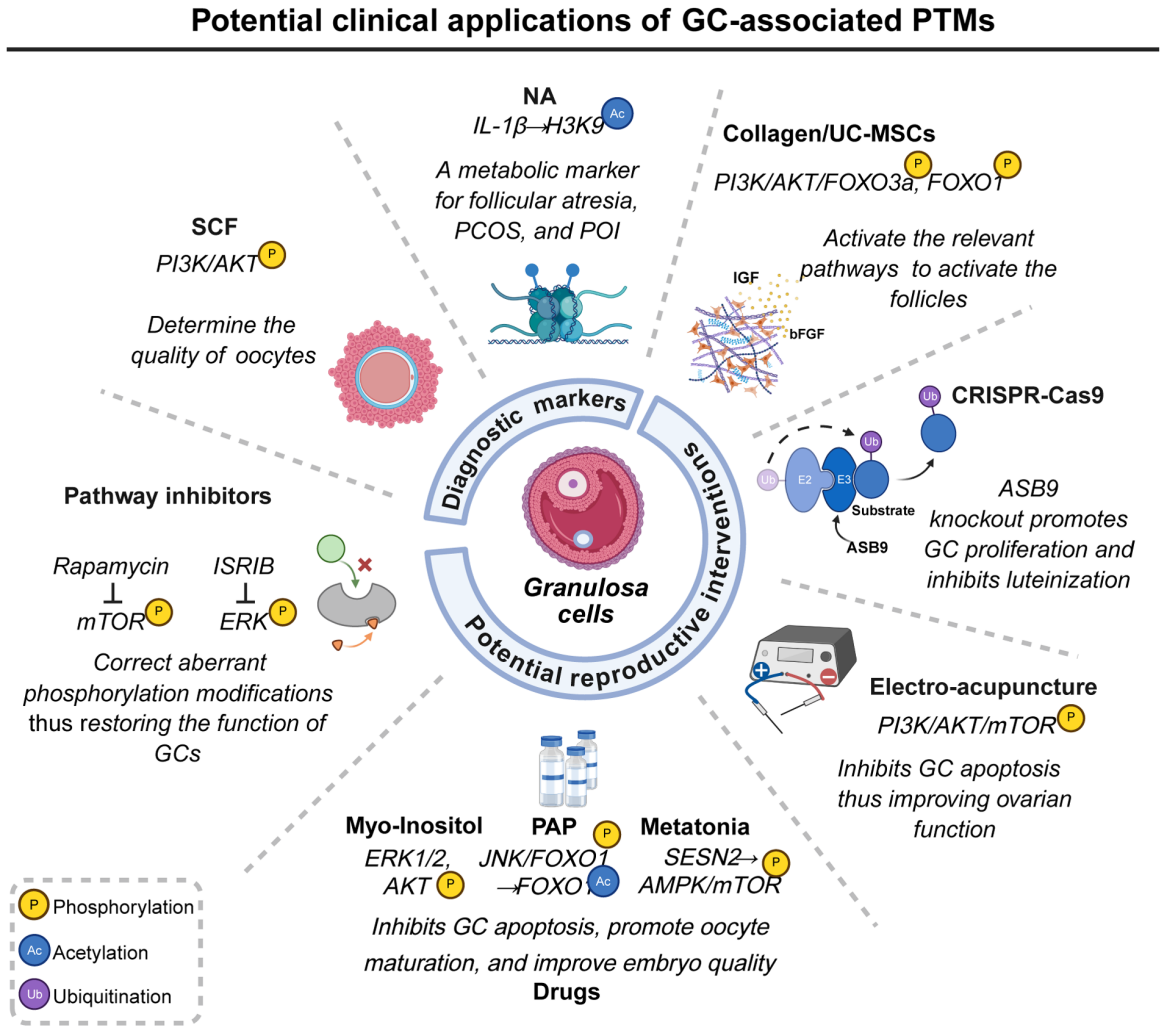
Potential clinical applications of PTMs in GCs. The specific patterns of PTMs in GCs, including phosphorylation, acetylation and ubiquitination, hold promise as novel biomarkers for diagnosing ovarian disorders and as potential. (Created in BioRender.com). PTM, post-translational modification; GCs, granulosa cells; NA, nervonic acid; SCF, stem cell factor; PAP, periplaneta americana peptide; UC-MSCs, umbilical cord mesenchymal stem cells; ISRIB, integrated stress response inhibitor; PCOS, polycystic ovary syndrome; POI, premature ovarian insufficiency; IGF, insulin-like growth factor; bFGF, basic fibroblast growth factor.

PTMs carry out key roles in follicular function by dynamically regulating protein activity, localization and interaction networks (32). Proteomic advances have enabled systematic profiling of PTM alterations in GCs linked to reproductive diseases, offering mechanistic insights into infertility (Fig. 2). These PTM patterns, particularly phosphorylation, acetylation, and ubiquitination, hold clinical potential as diagnostic biomarkers and therapeutic targets (154-156).

### Diagnostic candidate markers for GC-associated PTM

PTMs, which critically regulate the function of GCs, demonstrate notable potential as precise diagnostic biomarkers for female reproductive disorders (156-158).

In GCs, the stem cell factor (SCF) stimulates the activation and maturation of primordial follicles and enhances oocyte quality through modulation of the PI3K/AKT signaling pathway. Additionally, SCF in follicular fluid regulates oxidative stress and facilitates bidirectional communication between the oocyte and GCs. Given its multifaceted roles, SCF is recognized not only as a potential therapeutic target for conditions such as ovarian aging, infertility due to poor ovarian response and compromised oocyte quality but also as a non-invasive biomarker for assessing oocyte maturity and predicting pregnancy outcomes, particularly under antiretroviral therapy (155,159).

Nervonic acid (NA), primarily involved in sphingolipid metabolism and cell membrane structure formation (160), exhibits dual effects on GCs. Aberrant accumulation of NA markedly alters H3K9 acetylation through two mechanisms. Firstly, NA upregulates the deacetylase SIRT6, markedly reducing H3K9ac levels and transcriptionally repressing key steroidogenic genes, thereby disrupting estradiol synthesis and impairing luteal function. Secondly, NA enhances the recruitment of the transcription factor activator protein-1 (AP-1) to the IL-1β promoter region, specifically increasing H3K9ac levels at this site, which promotes the overexpression of IL-1β and exacerbates the ovarian inflammatory environment. Consequently, serum levels of NA may serve as valuable metabolic biomarkers for reproductive disorders such as PCOS, POI and follicular atresia (161).

The protein p27 (p27Kip1), a cyclin-dependent kinase inhibitor, induces G_1_/S arrest, suppresses GCs proliferation and triggers apoptosis, thus accelerating follicular atresia. Its expression is inversely associated with follicular survival and is considered as an indicator of ovarian reserve. Therefore, elevated levels of p27 in ovarian tissue or serum may serve as a non-invasive biomarker of POF, reflecting arrested follicular development and a declining reproductive capacity (162,163).

### Potential reproductive interventions for GC-associated PTMs

Precise modulation of targeted PTMs presents an innovative strategy for enhancing the follicular microenvironment.

A three-dimensional biomaterial, collagen/umbilical cord mesenchymal stem cells (UC-MSCs), has been developed. This biomaterial embeds UC-MSCs within a collagen scaffold, replicating the physicochemical properties of the native extracellular matrix. This configuration markedly enhances UC-MSC adhesion, proliferation and paracrine secretion, thereby improving the targeting efficiency and sustainability of stem cell-based therapies. Through paracrine signaling, collagen/UC-MSCs secrete insulin like growth factor and basic fibroblast growth factor, which activate the PI3K-AKT pathway in GCs. This activation leads to the phosphorylation of FOXO3a and FOXO1, facilitating their nuclear export and mitigating their inhibitory impact on primordial follicle activation. This mechanism rejuvenates the ovarian niche and provides a novel therapeutic opportunity for patients with POF (164).

CRISPR-Cas9 technology has emerged as a potent tool for epigenetic editing. ASB9, identified as a specific substrate recognition component of the E3 ligase, is differentially expressed in the GCs of ovulatory follicles. Previous studies employing CRISPR-Cas9-mediated ASB9 knockout have demonstrated an increase in GC number, providing robust evidence for the role of ASB9 as a regulator of GC function that limits GC proliferation and contributes to GC luteinization (154,165).

In conclusion, PTM-targeted interventions in GCs meld molecular mechanisms with clinical innovation, defining the forefront of reproductive medicine. Future research should focus on rigorous translational and clinical validation to ensure the safety and efficacy of these precision strategies for treating infertility.

## 5. Perspective

Infertility is increasingly recognized as a considerable global challenge affecting female reproductive health (166). As a fundamental regulatory mechanism of protein function, PTMs meticulously orchestrate protein networks within GCs and carry out a key role in reproductive pathologies. Emerging evidence suggests that PTM dysregulation in GCs is a key determinant in the pathogenesis of female infertility, offering novel mechanistic insights and therapeutic targets for clinical intervention. Notably, with the rise in global environmental pollution, exposure to toxins such as heavy metals, radiation and hazardous chemicals may disrupt physiological PTMs in GCs, leading to autophagy or apoptosis of these cells, ultimately exacerbating infertility (43,167,168). However, the molecular mechanisms underlying the interactions between environmental factors and reproductive health remain elusive, representing a notable area of scientific and clinical interest for the prevention and treatment of infertility amid increasing environmental pollution.

As the primary treatment modality for infertility, the long-term safety of ART necessitates further evaluation. Current evidence suggests that offspring conceived via ART may be at increased risk for rare imprinting disorders, potentially associated with aberrant epigenetic reprogramming in gametes or embryos (169). With the widespread adoption of ART, understanding its intergenerational health impacts is imperative. Notably, dynamic PTMs in GCs during ART have been shown to markedly influence the success of clinical pregnancies (30). However, few studies have explored whether *in vitro* manipulations disrupt embryonic epigenetic programming via PTM dysregulation in GCs. Thus, elucidating the molecular causality in the 'ART/PTM remodeling/embryonic development' pathway is crucial for optimizing ART procedures and ensuring intergenerational health.

Although targeted PTM therapy has achieved clinical success in oncology (170-173), its application in reproductive medicine remains in its early stages. While considerable progress has been made in mapping the PTM landscape of GCs, clinical application is constrained by several persistent challenges. Current evidence relies heavily on preclinical models, necessitating validation in large-scale human cohorts to establish pathological relevance. Additionally, the inherent dynamism and precisely timed fluctuations of PTMs across the follicular developmental continuum present a fundamental challenge for therapeutic targeting (87). Moreover, effective and ovary-specific drug delivery remains a pronounced technological barrier, requiring improved precision, kinetics and biocompatibility of delivery systems to minimize off-target effects (174,175). Future research should focus more on transitioning from individualized single-omics approaches to multi-omics strategies. Building a comprehensive PTM-omics database for GCs and associating it with large-scale clinical data will provide a key foundation for facilitating high-throughput drug screening (45,156,176).

In conclusion, the present review systematically assessed the dynamic regulatory networks of both canonical and novel PTMs in the proliferation, differentiation, apoptosis and hormone synthesis of GCs. It elucidates the pathological mechanisms of aberrant PTMs in female infertility, and comprehensively assesses the translational medical value of targeted PTM therapy for GCs. By integrating epigenetic regulation with clinical applications, this work aims to provide novel insights into precision diagnosis and treatment strategies for female infertility.

## Figures and Tables

**Table I:** Effects of phosphorylation on GCs.

Authors, year	Protein	Species	Mechanism	Effect	(Refs.)
Yang *et al*, 2023	ERK	Geese	FGF12-ERK	Promotes proliferation and inhibits apoptosis of GCs.	(44)
Zhao *et al*, 2023		Geese	MEK/ERK-BCL2	During the brood period, the downregulation of MEK reduces ERK phosphorylation and inhibits BCL2 expression, thereby inducing apoptosis in GCs.	(45)
Huang *et al*, 2022		KGN	HB-EGF/cAMP-PKA-ERK	Promotes hormone oversecretion, which subsequently impairs energy metabolism and triggers apoptosis in GCs	(41)
Liu *et al*, 2019		Porcine	pBD3/ERK1/2/Cyclin D1	Promotes GC proliferation and migration, which is mediated through ERK1/2 pathway activation and associated cyclin D1/PCNA upregulation.	(46)
Pan *et al*, 2022		Porcine	miR-574/TIMP3/ERK1/2	Promotes estradiol production in GCs by targeting TIMP3 and suppressing ERK1/2 phosphorylation.	(47)
Cottom *et al*, 2003		Sprague-Dawley rats	FSH/PKA/100-kDa PTP/ERK	Activates ERK signaling in GCs by relieving its tonic inhibition, thereby promoting downstream nuclear responses essential for follicle development.	(48)
Yan *et al*, 2023	AKT	Mice, KGN	MIGA1,2/AKT/Hippo-YAP1	Promotes GC proliferation by activating AKT and modulating the Hippo-YAP1 signaling axis.	(49)
Xue *et al*, 2024		Mice	PI3K/AKT	Induces apoptosis, autophagy and steroidogenesis disruption in GCs, thereby impairing oocyte quality and female fertility by inhibiting the PI3K/AKT pathway.	(43)
Song *et al*, 2023		Sprague-Dawley rats	SMAD3/AKT/MDM2/P53	Inhibits apoptosis and improves the activity of GCs, thereby ameliorating premature ovarian insufficiency.	(50)
Alam *et al*, 2004		Sprague-Dawley rats	FSH/PI3K/AKT/Rheb/mTOR	Induces GC differentiation and promotes the expression of follicular differentiation markers via HIF-1 activation.	(51)
Hu *et al*, 2020		Mice	PI3K/AKT/mTOR	Inhibits apoptosis and enhances estrogen production in GCs.	(52)
Zhang *et al*, 2024		Mice	cAMP/PI3K/AKT	Promotes primordial follicle activation by transmitting cAMP to the oocyte via gap junctions, thereby supporting follicle development.	(53)
Tong *et al*, 2022		PCOS patients	PI3K/AKT/mTOR	Insulin resistance contributes to the pathogenesis of PCOS by modulating GC autophagy and apoptosis.	(54)
Wu *et al*, 2025	CREB	Mice	FSH/cAMP/CREB	Promotes proliferation and differentiation of ovarian GCs through the synergistic action of CREB lactylation and phosphorylation, thereby driving follicular development.	(55)
Lanfranchi *et al*, 2022		Mice	cJUN-CREB-CBP/STAR	Regulates STAR expression and steroidogenesis, particularly under low oxygen conditions in GCs.	(56)
Zhang *et al*, 2022	mTOR	Mice	AMPK/mTOR	Enhances glycolysis in GCs to promote primordial follicle activation	(57)
Wu *et al*, 2023		Porcine	AMPK/mTOR	Ameliorates serum deprivation-induced apoptosis in porcine GCs by enhancing autophagy and activating PARylation signaling, thereby promoting cell survival and potentially inhibiting follicular atresia.	(58)
Lin *et al*, 2021		Mice, KGN	AMPK/mTOR/ULK1	Induces autophagy and reduces GC numbers and hormone production, leading to abnormal follicular development.	(59)
Liu *et al*, 2023		Porcine	ERβ/mTOR	PHB2 induces autophagy in porcine ovarian GCs, contributing to follicular atresia.	(60)
Liu *et al*, 2023		KGN	PI3K/AKT/mTOR	Promotes GC proliferation and reduces apoptosis by enhancing autophagy.	(61)
Tang *et al*, 2021		Bovine	AKT/mTOR	Induces autophagy and reduces cell viability in bovine GCs at high doses.	(62)
Hu *et al*, 2025		KGN, DOR mice	LIF/mTOR	Alleviates oxidative stress, improves mitochondrial function and inhibits apoptosis and ferroptosis in GCs under DOR conditions	(63)
Ji *et al*, 2024		KGN, Sprague-Dawley rats	RPL11-MDM2/p53/mTOR	Activates autophagy in GCs under PCOS conditions via nucleolar stress response, which disrupts GC function and contributes to abnormal follicle development and hyperandrogenism.	(64)
Li *et al*, 2024	FOXO1	Bovine	FOXO1-STAT3/AKT1-FOXO3	FOXO1 promotes GC apoptosis under hypoxic conditions through a FOXO3-dependent autophagy pathway.	(65)
Sun *et al*, 2020		Mice	GLP-1/GLP-1R/FOXO1	Regulates proliferation and anti-apoptosis of ovarian PCOS-associated GCs, which are involved in follicle development.	(66)
Lei *et al*, 2023		Bovine	PI3K/AKT/FOXO1	Induces oxidative stress, promotes apoptosis and disrupts steroid hormone synthesis in bovine GCs.	(67)
Yan *et al*, 2025		Hen	PI3K/AKT/FOXO1	Promotes GC proliferation and inhibits apoptosis by inducing FOXO1 phosphorylation and nuclear exclusion, thereby supporting follicle selection and growth.	(68)
Yang *et al*, 2020	JNK and p38 MAPK	Porcine	ERK1/2, JNK	Dynamically regulates GC apoptosis during follicular atresia by modulating opposite phosphorylation patterns of BimEL at Ser65 and Thr112 sites.	(69)
Ling *et al*, 2017		Sprague Dawley rats	MAPK (ERK↑, JNK/p38↓)	Protects rat GCs from oxidative stress-induced apoptosis by alleviating ROS damage and regulating apoptotic pathways.	(70)
Przygrodzka *et al*, 2021	LH/PKA	Bovine	LH-PKA/AMPK	LH and AMPK differentially regulate progesterone synthesis in luteal cells by oppositely modulating hormone-sensitive lipase activity and cholesterol availability.	(71)
Hou *et al*, 2010		Bovine	LH/PKA/cAMP/GSK3β-AMPK/mTOR	Stimulates mTOR signaling and progesterone synthesis through pathways independent of AKT and MAPK, involving inhibition of GSK3β and AMPK.	(72)
Tsui *et al*, 2023	DRP1	Mice	Energy metabolism reprogramming, regulation of mitochondrial dynamic imbalance	Protects female GCs from ROS-induced oxeiptosis by improving mitochondrial function and reprogramming cellular energy metabolism.	(73)
Zareifard *et al*, 2023	JAK/STAT	Bovine	JAK3/STAT3	Promotes GC proliferation and steroidogenesis	(74)

ERK, extracellular signal-regulated kinases; FGF12, fibroblast growth factor 12; GCs, granulosa cells; MEK, mitogen-activated protein kinase kinase; BCL2, B-cell lymphoma 2; KGN, human granulosa cell tumor cell line; HB-EGF, heparin-binding epidermal growth factor; cAMP, cyclic adenosine monophosphate; PKA, protein kinase A; pBD3, porcine β-defensin 3; PCNA, proliferating cell nuclear antigen; miR-574, microRNA-574; TIMP3, tissue inhibitor of metalloproteinases 3; FSH, follicle-stimulating hormone; PTP, protein tyrosine phosphatase; MIGA1,2, mitoguardin 1 and 2; YAP1, yes-associated protein 1; PI3K, phosphoinositide 3-kinase; SMAD3, SMAD family member 3; MDM2, mouse double minute 2 homolog; Rheb, ras homolog enriched in brain; mTOR, mechanistic target of rapamycin; HIF-1, hypoxia-inducible factor 1; PCOS, polycystic ovary syndrome; CREB, cAMP response element-binding protein; cJUN, Jun protooncogene; CBP, CREB-binding protein; STAR, steroidogenic acute regulatory protein; AMPK, AMP-activated protein kinase; PARylation, Poly(ADP-Ribosyl)ation; ULK1, unc-51 like autophagy activating kinase 1; ERβ, estrogen receptor β; PHB2, prohibitin 2; LIF, leukemia inhibitory factor; DOR, diminished ovarian reserve; RPL11, ribosomal protein L11; FOXO1, forkhead box O1; GLP-1, glucagon-like peptide-1; GLP-1R, glucagon-like peptide-1 receptor; BimEL, Bcl-2 interacting mediator of cell death extra long; JNK, c-Jun N-terminal kinase; MAPK, mitogen-activated protein kinase; ROS, reactive oxygen species; LH, luteinizing hormone; GSK3β, glycogen synthase kinase 3 β; Drp1, dynamin-related protein 1; JAK, Janus kinase; STAT, signal transducer and activator of transcription.

**Table II:** Effects of methylation on GCs.

Protein (loci)	Species	Mechanism	Effect	(Refs.)
H3K4me3	Cxxc1fl/fl mice	PI3K/AKT	Deficiency indirectly impairs proliferation and apoptosis of GCs, inducing follicular atresia,	(81)
H3K4me3, H3K9me3, H3K27me3 (*StAR* promoter region)	Sprague-Dawley rat	ERK-1/2	Induction of ovulation by hCG luteinizes GCs, favoring subsequent corpus luteum formation.	(82,83)
H3K4me3, H3K9me3, H3K27me3 (*Cyp19a1* promoter region)	Sprague-Dawley rat	Regulation of transcription factor C/EBPβ and phosphorylated CREB	Facilitates progesterone synthesis by luteinized GCs after the LH surge.	(83)
H3K9me2 (*Cyp19a1* PII, PI.4 promoter region)	Patients with EMS-infertility receiving IVF/ICSI	/	Low aromatase expression in Patients with EMS-infertility.	(84)
H3K9me2 (*Cyp19a1* PII promoter region)	Patients with PCOS receiving IVF/ICSI	/	Regulates aromatase expression; abnormal expression results in defective folliculogenesis.	(85)
H3K4me3, H3K9me3, H3K27me3 (*Cyp11a1* proximal promoter region)	Sprague-Dawley rat	PKA/cAMP	Chromatin spatial restructuring for progesterone formation.	(86)
H3K4me, H3K4me2, H3K4me3	Porcine	/	H3K4 distribution patterns change with follicular development and can be used as an epigenetic marker.	(87)
H3K4me3, H3K9me3, H3K27me3 (repressor α proximal promoter region)	Sprague-Dawley rats	DNMT3a silences inhibin α	Regulation of spatiotemporal expression of inhibin α and ovulation and luteinization.	(15,88)
H3K4me3, H3K9me3, H3K27me3 (C/EBPβ binding region in VEGF promoter)	Sprague-Dawley rats/KGN	regulation by C/EBPβ-binding promoter	Regulation of angiogenesis during luteinization.	(89)
H3K36me1/2/3	Bovine	Increased expression of PRC2 components (EZH2 and SUZ12)	Inhibition proliferation and promotion of apoptosis of GCs.	(90)
H3K27me3	Porcine	Transcriptional repression of *RUNX1*	Determines hormone synthesis, apoptosis and proliferation in the pGC microenvironment.	(91)
H3K9me2/3 (*FMR1* promoter and exon 1 region)	Patients with DOR	Affects the expression of *FMR1*	Associated with primordial follicle activation and diminished ovarian reserve function.	(92,93)
H3K4me2/3	Porcine	Influences the cell cycle through BPTF	Cell cycle arrest in G_2_/M phase, which promotes GC apoptosis.	(94)
H3K4me1/2/3, H3K9me1/2/3	Caprine, Porcine, Bovine	Upregulation of MTF1, demethylation enzymes KDM4B, KDM5B and KDM5C regulation	Inhibits DNA damage and promotes the cell cycle in GCs (promotes proliferation, inhibits apoptosis).	(95-97)
HnRNPA1	Mice	IRES-dependent translation of *Wt1* mRNA, a key transcription factor promoting follicular development	Regulation of GC differentiation and follicular development.	(98)

H3, histone H3; K, lysine; me, methylation; me1, mono-methylation; me2, di-methylation; me3, tri-methylation; GCs, granulosa cells; Cxxc1 CXXC finger protein 1; PI3K, phosphoinositide 3-kinase; AKT, protein kinase B; ERK-1/2, extracellular signal-regulated kinase 1/2; StAR, steroidogenic acute regulatory protein; hCG, human chorionic gonadotropin; Cyp19A1, cytochrome P450 family 19 subfamily A member 1 (Aromatase); C/EBPβ, CCAAT/enhancer binding protein β; CREB, cAMP response element-binding protein; LH, luteinizing hormone; Cyp11a1, cytochrome P450 family 11 subfamily A member 1; CYP, cytochrome P450; EMS, endometriosis; IVF, in vitro fertilization; ICSI, intracytoplasmic sperm injection; PCOS, polycystic ovary syndrome; PKA, protein kinase A; cAMP, cyclic adenosine monophosphate; DNMT3a, DNA methyltransferase 3 α; VEGF, vascular endothelial growth factor; PRC2, polycomb repressive complex 2; EZH2, enhancer of zeste homolog 2; SUZ12, SUZ12 polycomb repressive complex 2 subunit; RUNX1, runt related transcription factor 1; pGC, preantral granulosa cell; FMR1, fragile X messenger ribonucleoprotein 1; DOR, diminished ovarian reserve; BPTF, bromodomain PHD finger transcription factor; MTF1, metal regulatory transcription factor 1; KDM4B, lysine demethylase 4B; KDM5B, lysine demethylase 5B; KDM5C, lysine demethylase 5C; HnRNPA1, heterogeneous nuclear ribonucleoprotein A1; IRES, internal ribosome entry site; Wt1, Wilms tumor 1.

**Table III:** Effects of acetylation on GCs.

Protein (loci)	Species	Mechanism	Effect	(Refs.)
H3K27	Mice	Erase and re-establishment of acetylation.	Promotes transcription of relevant genes.	(106)
H3K9	Human	Activation of PPARγ and PGC1α pathways.	The former promotes steroidogenesis, the latter attenuates oxidative stress.	(107)
Histones of the *Cox1* promoter region	Mice	Nicotine increases levels of HDAC3 and decreases levels of acetylation in the COX1 promoter region.	Reduces PGE2 secretion, leading to apoptosis and autophagy in GCs, impeding follicle maturation.	(108)
H3K9	Rats/human	Chromatin remodeling triggers the expression of the CYP19A1 or the repressor α gene.	Promotes follicular development and estrogen synthesis.	(15,85)
H3, H4	Rats	Chromatin remodeling triggers StAR expression and represses CYP19A1.	Shifts from estrogen to progesterone synthesis in GCs undergoing luteinization.	(20)
H2BK5, H3K9	Mice	ERK1/2 and CITED4-CBP promote chromatin remodeling of relevant genes.	Promotes ovulation.	(109)
ACAT1 K-174	Human	High acetylation inhibits enzyme activity and reduces acetyl-CoA and succinate production.	Impairs energy supply to oocytes.	(110)
DNMT1 K1118, K1120, K1122, K1124	Mice	Acetylation of this region disrupts its interaction with USP7	Leads to DNA hypomethylation and senescence, causing GC cell cycle arrest.	(111)
FOXO1	Mice	Oxidative stress upregulates miR-181a, and miR-181a, which downregulates the deacetylase SIRT1 and reduces FOXO1 deacetylation.	Promotes FOXO1 nuclear translocation, activates anti-apoptotic genes and induces apoptosis in GCs.	(112)
FOXO1	Porcine	JNK/FOXO1 pathway.	Inhibits apoptosis induced by oxidative stress in porcine ovarian GCs.	(113)
FOXO1	Mice	HAT1 promotes FOXO1 nuclear translocation, binds to the AREG promoter region and increases AREG expression.	AREG induces meiotic resumption in oocytes.	(114)
P53	Human	SIRT1 induces p53 deacetylation, upregulating PARP and PUMA.	Mediates apoptosis in GCs, impairing oocyte quality and ovarian function.	(115)
P53	Human	TOPK inhibition decreases SIRT1 expression and increases p53 acetylation levels.	Elevated p53 acetylation enhances its activity and upregulates pro-apoptotic gene expression, increasing apoptosis in GCs.	(116)
P53-K382	Human	Inhibition of SIRT1 activity or expression.	Enhances p53 transcriptional activity, leading to apoptosis in GCs.	(117)

H3, histone H3; K, lysine; H4, histone H4; H2B, histone H2B; ac, acetylation; PPARγ, peroxisome proliferator-activated receptor γ; PGC1α, PPARγ coactivator 1α; Cox1, cyclooxygenase 1; HDAC3, histone deacetylase 3; PGE2, prostaglandin E2; GCs, granulosa cells; CYP19A1, cytochrome P450 family 19 subfamily A member 1; StAR, steroidogenic acute regulatory protein; ERK1/2, extracellular signal-regulated kinases 1/2; CITED4, Cbp/p300-interacting transactivator 4; CBP, CREB-binding protein; ACAT1, Acetyl-CoA acetyltransferase 1; CoA, coenzyme A; DNMT1, DNA methyltransferase 1; USP7, ubiquitin specific peptidase 7; miR-181a, microRNA-181a; SIRT1, sirtuin 1; HAT1, histone acetyltransferase 1; AREG, amphiregulin; PARP, poly (ADP-ribose) polymerase; PUMA, p53 upregulated modulator of apoptosis; TOPK, T-LAK cell-originated protein kinase.

**Table IV:** Effects of ubiquitination on GCs.

Protein (loci)	Species	Mechanism	Effect	(Refs.)
DRP1	Rats	DRP1 initiates mitochondrial fission. E3 ligase SYVN1 induces the degradation of DRP1	SYVN1 inhibits apoptosis and mitochon drial fission by promoting the degradation of DRP1	(125)
NCOA4	Human/Mice	*Cry1* depletion reduces the expression of HERC3 and decreases NCOA4 degradation	Promotes ferritin delivery to lysosomes and degradation, increases intracellular iron levels, and induces cellular senescence	(126)
Ku70/H2AX/DDB1/RNF168	Human/Mice	USP14 deubiquitinates proteins, leading to a reduced ability of Ku70 to bind to the DSB site and preventing the advance of NHEJ	USP14 overexpression reduces NHEJ, downregulates DDR-related proteins, increases DNA damage, and promotes cellular senescence	(127)
K4, K64 in VDAC2	Porcine	UCHL1 removes ubiquitin chains on VDAC2 to prevent their degradation.	VDAC2 enhances cholesterol transport to the inner mitochondrial membrane and promotes E2 synthesis	(128)
Androgen receptor	Human	PGK1 inhibits AR ubiquitination levels, promotes its nuclear translocation, and increases its stability	Elevated Nuclear AR Levels Lead to AR Overtranscription and Overexpression of Key Ovulatory Genes Associated with Hyperandrogenemia and Abnormal Folliculogenesis in PCOS	(129)
GPX4	Human Mice	NEDD4L directly interacts with GPX4 and promotes its degradation	Promotes GC ferroptosis	(130)
ATG7	Chicken	USP13 inhibits ATG7 ubiquitination and degradation in GCs	ATG7 promotes ferritin degradation via autophagy activation, enhancing Fe2+ accumulation in GCs and triggering ferroptosis	(131)
MCM5	Human Mice	PFAP1 directly binds to MCM5 and inhibits its ubiquitination and degradation	Enhances MCM5 activity and its DNA replication-associated functions to support GCs' survival, proliferation, and follicular development	(132)
TGFβR2/SMAD4	Porcine	TGF-β	Inhibits apoptosis of GCs and maintains ovarian function	(133)
BimEL	Porcine	ERK1/2	Causes degradation of BimEL, inhibits apoptosis of GCs	(134)

DRP1, dynamin-related protein 1; SYVN1, synoviolin 1 (E3 ubiquitin ligase); NCOA4, nuclear receptor coactivator 4; Cry1, cryptochrome circadian regulator 1; HERC3, HECT and RLD domain containing E3 Ubiquitin protein ligase 3; Ku70, XRCC6, X-ray repair cross complementing protein 6; H2AX, H2A histone family member X; DDB1, damage specific DNA binding protein 1; RNF168, ring finger protein 168; USP14, ubiquitin specific peptidase 14; DSB, double-strand break; NHEJ, non-homologous end joining; DDR, DNA damage response; K, Lysine; VDAC2, voltage-dependent anion channel 2; UCHL1, ubiquitin C-terminal hydrolase L1; E2, estradiol; AR, androgen receptor; PGK1, phosphoglycerate kinase 1; PCOS, polycystic ovary syndrome; GPX4, glutathione peroxidase 4; NEDD4L, neuronal precursor cells expressed developmentally down-regulated 4-like, NEDD4 like E3 ubiquitin protein ligase; ATG7, autophagy related 7; USP13, ubiquitin specific peptidase 13; Fe2+, ferrous ion; MCM5, minichromosome maintenance complex component 5; PFAP1, primordial follicle activation peptide 1; TGFβR2, transforming growth factor β receptor 2; SMAD4, SMAD family member 4; TGF-β, transforming growth factor β; BimEL, Bcl-2-like protein 11 extra long; ERK1/2, extracellular signal-regulated kinases 1/2.

**Table V:** effects of novel post-translational modification on GCs.

PTMs	Protein (loci)	Mechanism	Effect	(Refs.)
Lactylation	H3K18	In hypoxia, hCG promotes lactate production, activates EP300/CBP and catalyzes H3K18 lactylation.	Promotes luteinization of GCs and increases progesterone synthesis. Accelerates the conversion of cholesterol to progesterone.	(22)
Crotonylation	ANXA2	EP300-ANXA2-EGFR.	Promotes proliferation and inhibits apoptosis of cumulus cells, thereby affecting the meiotic resumption and maturation of oocytes.	(23)
Neddylation	Cullin	MLN4924 inhibits NAE (NEDD8-activating enzyme) and blocks Cullin protein mimicry.	Inactivates CRL and blocks the cell cycle inhibitory protein accumulation, promoting cell proliferation.	(19)
O-GlcNAcylation	Serine/Threonine	OGT and OGA mediate dynamic modifications of S and T.	O-glcNAc imbalance leads to a decrease in mitochondrial membrane potential, promotes apoptosis and impairs glucose metabolism.	(138)
Succinylation	Lysine	Succinyl-CoA mediates lysine residue modification, changing the charge from +1 to -1.	Promotes apoptosis of GCs, increases follicular atresia, decreases ovarian reserve hormone (AMH, E2) levels and upregulates p21, a marker of aging.	(25)

PTMs, post-translational modifications, H3, histone H3; K, lysine; hCG, human chorionic gonadotropin; EP300, E1A binding protein P300; CBP, CREB binding protein; GCs, granulosa cells; ANXA2, annexin A2; Kcr, crotonylation; EGFR, epidermal growth factor receptor; MLN4924, pevonedistat; NAE, NEDD8-activating enzyme; NEDD8, neural precursor cell expressed developmentally downregulated protein 8; CRL, cullin-RING ligases; CoA, coenzyme A; AMH, anti-Mullerian hormone; E2, estradiol; p21, cyclin dependent kinase inhibitor 1A; OGT, O-linked N-acetylglucosamine transferase; OGA, O-GlcNAcase; O-GlcNAc, O-linked N-acetylglucosamine; S, serine; T, threonine.

## Data Availability

Not applicable.
